# Effects of Different Nitrogen Forms and Competitive Treatments on the Growth and Antioxidant System of *Wedelia trilobata* and *Wedelia chinensis* Under High Nitrogen Concentrations

**DOI:** 10.3389/fpls.2022.851099

**Published:** 2022-03-24

**Authors:** Ping Huang, Fangyuan Shen, Adeel Abbas, Hao Wang, Yizhou Du, Daolin Du, Sadam Hussain, Talha Javed, Saud Alamri

**Affiliations:** ^1^School of Environment and Safety Engineering, Institute of Environment and Ecology, Jiangsu University, Zhenjiang, China; ^2^Faculty of Engineering, School of Computer Science, University of Sydney, Sydney, NSW, Australia; ^3^College of Agronomy, Northwest A&F University, Yangling, China; ^4^Department of Agronomy, University of Agriculture Faisalabad, Faisalabad, Pakistan; ^5^College of Agriculture, Fujian Agriculture and Forestry University, Fuzhou, China; ^6^Department of Botany and Microbiology, College of Science, King Saud University, Riyadh, Saudi Arabia

**Keywords:** alien invasive plants, high N condition, N forms, competition, antioxidant enzymes

## Abstract

Nitrogen (N) is one of the essential nutrients for plant growth. Appropriate application of N can improve the N use efficiency (NUE) and significantly promote plants’ growth. However, under N toxic conditions, the relationship between the growth and antioxidant system of invasive plants under different N forms and competitive treatments is not fully understood. Therefore, in this study, the performance of invasive species *Wedelia trilobata* and its native species *Wedelia chinensis* was evaluated under two sets of N forms and ratios, namely, NH_4_^+^-N(AN)/NO_3_^–^-N(NN) = 2:1 and NH_4_^+^-N(AN)/NO_3_^–^-N(NN) = 1:2 along with two intraspecific and interspecific competitions under without N and high N level of 15 g N⋅m^–2^ year^–1^, respectively. Data regarding the growth indices, antioxidant enzyme activities, including peroxidase (POD) and catalase (CAT), malondialdehyde (MDA), and proline contents were determined. Results showed that for competitive treatments, growth status was better for interspecific competition than intraspecific competition. The plant biomass of *W. trilobata* was significantly higher than that of *W. chinensis*. N significantly promoted the plants’ growth in terms of leaf area and biomass yield, and the antioxidant enzyme activities were significantly increased under a high N treatment than that of the control. Among N forms/ratios, ammonium N (AN)/nitrate N (NN) = 2:1 significantly enhanced the enzyme activity, particularly in *W. trilobata*. Furthermore, for intraspecific competition, MDA contents of *W. trilobata* were significantly decreased compared to that of *W. chinensis*. In conclusion, our results showed that *W. trilobata* adapted well under competitive conditions through better growth and antioxidant defense system.

## Introduction

Under changing climatic conditions, the occurrence of environmental stresses along with changes in nitrogen (N) deposition is among the significant causes for limited agricultural production globally (Dong et al., 2020). N deposition, somehow, provides a new source of fertilizer for plant growth. An appropriate increase in N rate is beneficial to plant growth and developmental processes (Dong et al., 2020). However, if it is excessive, it can affect human health, change the biogeochemical cycle, change the structure and functions of an ecosystem, and may lead to species extinction (Hannah, 2021; Zhao et al., 2021). During the past 30 years, N deposition in China has increased about 60%; N is mainly absorbed by plants in organic and inorganic forms (Zhou et al., 2019; Guo et al., 2021). The plant roots can directly absorb and use inorganic N, mainly in the forms of ammonium N (AN) and nitrate N (NN) (Lu et al., 2021; Wang H. et al., 2021). However, plant sensitivity to these forms varies due to differences in species, genotypes, and soil conditions (Guo et al., 2019; Chalk and Smith, 2021). Compared with native species, invasive species usually have superior phenotypic plasticity, resource utilization efficiency, and better growth rates (Vaz-Pinto et al., 2014), which help invasive species survive even under stressful conditions. At present, the alien biological invasion has become a social, economic, and environmental problem globally (Renault et al., 2021). Therefore, controlling the invasion of alien species has become an urgent need (Shuvar and Korpita, 2021).

In recent years, N deposition, also called atmospheric deposition, has become a significant threat to soil processes and plant biodiversity (Wu et al., 2021). N is a major nutrient and an important constituent during the sustainable production period of plant growth (Shah et al., 2017a) and adequate supply associated with high photosynthetic activities (Shah et al., 2017b). Different plants show different responses and preferences to different N forms (Yousaf et al., 2016a; Qian et al., 2021). Under this situation, the study of N forms has gained much attention (He et al., 2021). Plants show various responses to different N forms and application rates. For example, Chen et al. (2020) studied the growth of citrus seedlings under different N rates and N forms and reported that its seedlings were susceptible to AN. Similarly, Zou et al. (2020) studied the growth traits of Moso bamboo (*Phyllostachys edulis*) under the application of different N forms and reported that AN significantly promoted the growth traits more than NN. In recently published reports, Shah et al. (2017a,2021a,b) studied different N rates under field conditions and reported that N rates non-significantly influenced the growth and physiological traits at the seedling stage; however, there was a significant influence at squaring stage where maximum values were recorded for low N rate, depending on the planting densities.

Furthermore, it is well established that the application of different N forms greatly influences the absorption of heavy metals by crop plants. In this context, working with dwarf polish wheat, Cheng et al. (2020) studied the uptake and accumulation of cadmium (Cd) under the application of various N forms and reported that the addition of NH_4_^+^-N has not only promoted the growth but also reduced the uptake and accumulation of Cd than NO_3_^–^-N. Similarly, Emamverdian et al. (2015) and Bai et al. (2020) demonstrated that the addition of mixed N forms (NO_3_^–^ and NH_4_^+^) significantly facilitated the growth and adaptation through a better antioxidant defense mechanism in legume plants under heavy metal contamination in degraded mining areas. Working with chromium-exposed barley crop, Ali et al. (2013) studied the effect of N forms on photosynthesis and antioxidant system and found that the addition of NN significantly increased the photosynthesis and antioxidant enzyme activities and reduced the chromium content in tissues to enhance the ability of plants to grow well under stress environment. Similarly, de Souza Junior et al. (2019) have reported that the addition of N in the forms of NN and AN enhances the tolerance of *Tanzania guinea* grass to copper stress. N, one of the most important nutrients for plant growth, can influence the growing competition between the crop plants, including invasive species (Van Hezewijk et al., 2008), depending on the application rates and time of application (Razaq et al., 2017; Anas et al., 2020). The addition of N as nitric oxide enhances arsenic (As) tolerance in *Brassica juncea* by promoting its growth and reducing As uptake (Ahmad et al., 2021). Heavy metals are toxic to plants; however, the addition of N in different forms can enhance plant tolerance to heavy metals through an improved antioxidant defense system. However, it is also well established that high N can cause oxidative damage to crop plants (Chu et al., 2021). Therefore, the optimization of N rates, particularly when applying in different forms, is essential to reduce the damage caused by oxidative stress under high N (Chen et al., 2020). Furthermore, the impacts of various N rates were also studied for invasive plant species. For example, working with *Cynoglossum officinale* (L.), Van Hezewijk et al. (2008) reported that fertilization treatment increased the percentage of N by dry weight in the leaf tissue. In another study, Qing et al. (2011) reported that N addition significantly affected the individual traits of both invasive Jiangsu and native Georgia *Spartina alterniflora* populations, where invasive populations showed stronger responses to N addition in total biomass, number of leaves, total leaf area, and maximum culm height than native populations. In a recent study, Peng et al. (2019) evaluated the performance of invasive species *Solidago canadensis* and reported that N addition extended the leaf life span, increased the ramet height, and advanced the onset of inflorescence and flowering.

*Wedelia trilobata*, a creeping herb with wide adaptability, is a native plant to Central America and has invaded tropical and subtropical regions (Qi et al., 2014). During the late twentieth century, it was introduced to China as an ornamental plant; however, under a short period, it was recognized as an invasive species of the country (Li and Xie, 2002). It is now widely distributed in China’s Guangdong, Guangxi, and Fujian provinces. Previous studies on N deposition and N forms mainly focused on crop plants; however, only few studies are on invasive plants to study the growth traits (Chen et al., 2016). Still, no adequate study reported the effect of different N forms on the defense behavior of invasive species under high N. Therefore, for this study, we hypothesized that different N forms and plant combination patterns would enhance the success of invasive *W. trilobata* over native *Wedelia chinensis* due to better physiological response and improve growth traits. In this study, an exotic invasive plant *W. trilobata* was taken to analyze the internal mechanisms of plant invasion in terms of the plant antioxidant system. Our specific objectives were to (1) evaluate the effect of different N forms and ratios on plant growth and biomass distribution under high N condition and (2) the effect of N forms and ratios on antioxidant enzyme activities in invasive and native plant species under different competitive treatments.

## Materials and Methods

### Experimental Materials

**FIGURE 1:**
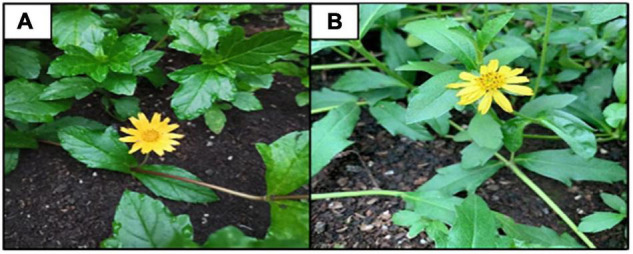
Plant species used for this experiment. **(A)** Invasive *Wedelia trilobata*. **(B)** Native *Wedelia chinensis*.

The invasive *W. trilobata* and native *W. chinensis* species were collected from Nanning City, Guangxi Province, and the greenhouse of the School of Environmental Sciences, Jiangsu University, respectively, and used for this experiment (Figure 1). The regenerated stem segments of *W. trilobata* and *W. chinensis* with robust and consistent growth (length and thickness) were selected, and two nodes were retained for each stem segment. Cuttings were cultured in 90 mm × 60 mm × 80 mm round plastic pots where two plants were cultivated in each pot. To ensure no nutrients in the substrate as far as possible and the air permeability of plant roots, the river sand has been screened, washed, dried, and sterilized with green zeolite (sand: green zeolite = 5:1) as the substrate needed in this experiment, and 300 g was weighed for each pot. The selected stem segments were cultured vertically in the same position in pots, irrigated with deionized water, and placed in the greenhouse at the School of Environmental Sciences, Jiangsu University. Upon reaching approximately 3 cm of height (after 4–5 days), the predefined AN/NN proportions were supplied.

### Experimental Method and Design

According to the data obtained by the National Nitrogen Deposition Monitoring Network, due to the rapid economic development in recent years, N deposition has become a severe issue throughout China. The total amount of N deposition is varied among various regions of the country. For example, in 2020, the total amount of N deposition in Jiangsu Province was reported as 10 g N⋅m^–2^⋅year^–1^, having an increasing rate of 0.6 g N⋅m^–2^⋅year^–1^ compared to the previous year. Taking into account the results of pre-experiments and the trend of N deposition in recent years, the N deposition concentration set in this experiment was 15 g N⋅m^–2^⋅year^–1^, and 0 g N⋅m^–2^⋅year^–1^ was kept as a control for comparison. The experiment was designed in a completely randomized block design under a factorial arrangement consisting of two forms and ratios, namely, AN/NN with ratios 2:1 and 1:2 in the nutrient solution, and two competitive modes, namely, intraspecific competition (two *W. trilobata* or two *W. chinensis* plants in each pot) and interspecific competition (one *W. trilobata* and one *W. chinensis* in each pot), with a total of eight replicates (three for growth, physiological, and nutritional assessments, and five for metabolic evaluations). The KNO_3_ and NH_4_NO_3_ salts were used for the AN/NN proportions. The preparation of N deposition solution refers to the protocol of Luo et al. (2019), and the ratio of solution with different N deposition levels is shown in Table 1. After the cultivation period, the experimental units (plant species) were cultured for 45 days with Hoagland’s nutrient solution, irrigated at every 5 days, where 40 ml of solution was applied each time, and the total N content was 15 g. The Hoagland’s nutrient solution without N was used as a control treatment in the experiment. Deionized water, as a supplement, was applied with an equal amount at 2–3 days intervals to ensure the plant’s normal growth.

**TABLE 1:** Solution ratios of different nitrogen deposition and nitrogen forms.

Nitrogen treatment (g m^–2^ year^–1^)	Treatment components	Concentration: AN:NN = 2:1 (mol L^–1^)	Concentration: AN:NN = 1:2 (mol L^–1^)
0	K_2_SO_4_	0.0075	
	KCl	0.0150	
15	(NH_4_)_2_SO_4_	0.0050	0.0025
	KNO_3_	0.0050	0.0100
	K_2_SO_4_	0.0025	0.0050
	KCl	0.0200	0.0100

*AN:NN, Ammonium nitrogen (AA) and nitrate-Nitrogen (NN).*

### Data Collection

Plants from each pot were harvested 45 days after cultivation. The aboveground parts (e.g., leaves and stem) and underground parts (e.g., roots) of both species *W. trilobata* and *W. chinensis* were separated, and the dry weights, leaf area, number of leaves, number of main roots, root length, number of lateral roots, chlorophyll content, leaf N content, chlorophyll fluorescence, and antioxidant system-related indicators were measured. The measurement of each indicator was repeated three times.

#### Plant Growth

The collected fresh leaves, stems, and roots were washed with clean water, placed at 105°C for 10 min, and then moved to an oven and dried at 65°C for 72 h. The leaf dry weight, stem dry weight, and root dry weight were weighed by using an analytical balance. The sum of these was used as total plant biomass, and the root-shoot ratio was the dry weight ratio of the underground parts to the aboveground parts. The number of leaves and roots was counted manually, and leaves area, plant height, and root length were accurately measured using the ImageJ software^1^ after taking pictures with a digital camera.

#### Plant Photosynthesis

Before harvesting plants, the chlorophyll content (e.g., the soil plant analysis development (SPAD) values) and leaf N contents in leaves were determined by using a portable chlorophyll meter, and the chlorophyll fluorescence-related indicators (i.e., *F*_*o*_, *F*_*m*_, *F*_*v*_, and *F*_*v*_/*F*_*m*_) were measured by using FluorPen handheld chlorophyll fluorescence meter. The data were collected from all plants of each treatment and repetition and then the average value was used for data analysis. Chlorophyll fluorescence is a technique used to describe the mechanism of photosynthesis. In this study, minimum fluorescence (*F*_*o*_) was recorded by turning on the light after 20 min of dark adaptation, and maximum fluorescence (*F*_*m*_) was measured after dark adaptation, followed by a saturation pulse with a short duration of 0.2–1.5 s. A variable fluorescence *F*_*v*_ (*F*_*v*_ = *F*_*m*_–*F*_*o*_; *F*_*v*_/*F*_*m*_) was the maximum photosynthetic efficiency of Photosystem II, reflecting the potential maximum light energy conversion efficiency of plants.

#### Oxidative Injuries

To determine the malondialdehyde (MDA) content, 0.5 g of leaves was ground in 2 ml of 10% trichloroacetic acid (TCA) in pestle and mortar. After centrifugation at 4,000 rpm for 10 min at 4°C, the supernatant was taken, followed by heating at 100°C for 20 min. The tubes were cooled quickly in an ice bath after heating. The absorbance was taken at wavelengths of 532, 600, and 450 nm by using a spectrophotometer. The MDA content was determined by using the following formula:

Malondialdehyde concentration (μmol L^–1^) = 6.45 (A532-A600)–0.56A450.

where A represents absorbance at the specified wavelength. Then, MDA content (μmol g^–1^) = *C* × *V*/(1,000 × *W*), where *V* denotes sample extraction liquid (ml) and *W* denotes the sample weight (g).

#### Antioxidant Enzyme Activities

Leaf catalase (CAT) and peroxidase (POD) activities: To determine the antioxidant activities, first, 0.1 g of plant leaves were weighed and ground with liquid N; after adding 2.5 ml of phosphate buffer (pH = 7.8), the solution was centrifuged at 4°C for 15 min at 10,000 rpm; and then the supernatant was used as an enzyme extract. Later, the ultraviolet spectrophotometry and guaiacol method were used to determine the activities according to the protocol developed by Wei et al. (2018). CAT activity was assayed in a mixed reaction system containing 0.1 ml of enzyme extract, 1 ml of hydrogen peroxide (H_2_O_2_) (0.3%), and 1.9 ml of deionized water. The reaction system was used to record the change of absorbance at 240 nm for 3 min (readings were taken with 30 s interval), and CAT activity (U g^–1^ FW⋅min) was expressed as the amount of enzyme that catalyzed the breakdown of H_2_O_2_ over time. Furthermore, POD activity was estimated by measuring the absorbance of a mixture comprising 0.05 ml of enzyme extract, 1 ml of H_2_O_2_, 1 ml of 50 mM phosphate buffer (pH = 7.0), and 0.95 ml of guaiacol (0.2%). Reading of each sample was measured for 3 min (readings were taken with 30 s interval), and POD activity (U g^–1^ FW⋅min) was expressed at 470 nm as the absorbance value increased over time.

Proline concentration: First, 0.1 g of plant leaves was taken into a test tube; after adding 5 ml of sulfosalicylic acid (30 g L^–1^), the mixture was reacted at 100°C for 10 min. After filtration, the mixture was used as proline extract for further analysis. The extract was reacted with ninhydrin (25 g L^–1^), glacial acetic acid, and phosphoric acid (6 mol L^–1^) at 100°C for 1 h. After that, concentrated toluene was added, and tubes were shaken for 20 s (Bates et al., 1973). The upper layer was analyzed in a spectrophotometer at 520 nm wavelength.

### Data Analysis

In this research, five physiological indices as the activities of CAT and POD, as well as proline and MDA contents were measured. SPSS (22.0) software was used to conduct univariate or multivariate ANOVA and correlation analysis for each factor. The software Origin 8.1 was used to chart the results of the statistical analysis of the data. A value of *p* < 0.05 was statistically significant for all biomarkers.

## Results

### Effects of Different N Forms and Competitive Treatments on Plant Growth

**FIGURE 2:**
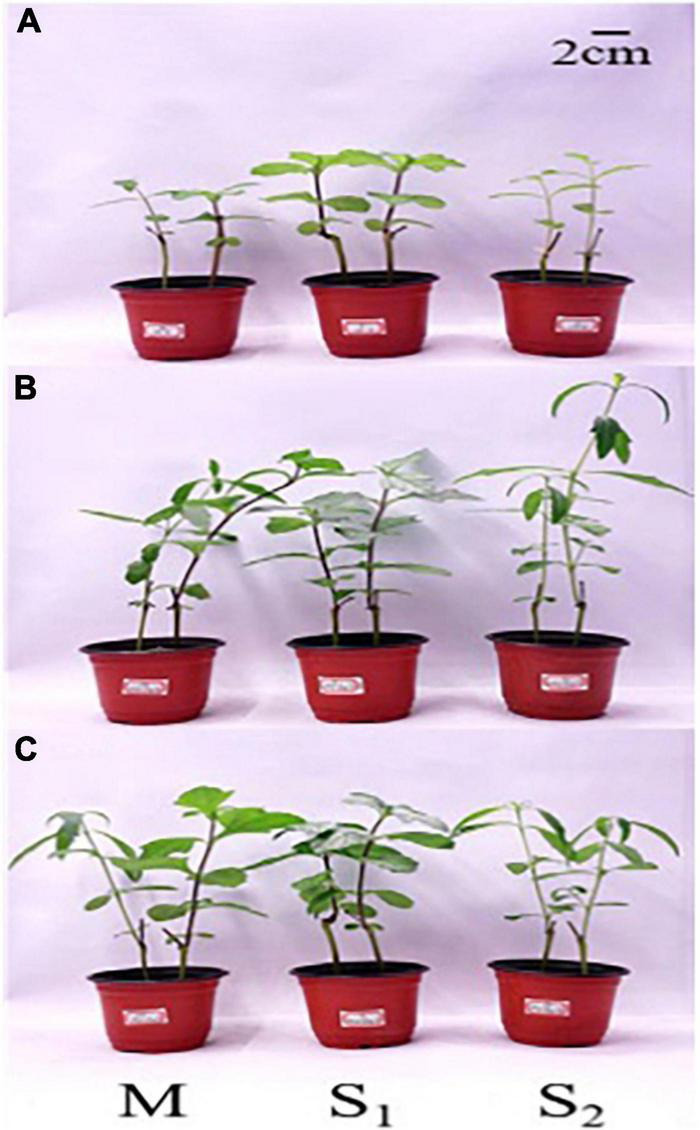
Effect of different nitrogen (N) forms and competitive treatments on plant growth under high N conditions. M, interspecific competition between *W. trilobata* and *W. chinensis*; AA, ammonium nitrogen; NN, nitrate nitrogen; S_1_, intraspecific competition of *W. trilobata*; S_2_, intraspecific competition of *W. chinensis*; **(A)** no nitrogen addition; **(B)** nitrogen form ratio AN:NN, 2:1 with total nitrogen amount of 15 g N⋅m^–2⋅^year^–1^; **(C)** nitrogen form ratio AN:NN, 1:2 with total nitrogen amount of 15 g N⋅m^–2⋅^year^–1^.

The effect of N forms and competitive treatments on plant growth is shown in Figure 2. Invasive species *W. trilobata* grows better than that of its native species *W. chinensis* under N addition, and competitive treatments had less effect on these species. When two species competed, the growth of both plants was better than the intraspecific competition, particularly in *W. trilobata*. Therefore, the competition provided a substantial invasion opportunity for *W. trilobata*. The effect of N forms on plant growth was not significant, but AN:NN = 2:1 was more favorable for plant growth, particularly for *W. chinensis*.

Under high N conditions (N concentration of 15 gm^–2^), the leaf area and total plant biomass were significantly higher, and the root-shoot ratio was significantly lower than those of the control treatment (Table 2). Under control condition (N concentration of 0 g m^–2^), the growth of plants had inhibited to a certain extent, especially in the aboveground parts; for underground parts, the absence of N promoted the development of plant roots when compared with high N condition. The competitive effect of tested species is mainly reflected in the development of the root system. Competition of two species promoted the development of the root system of both species (Table 2). The effect of N forms on plant growth was prompt for root development in *W. chinensis* under intraspecific competition. The ratio AN:NN = 1:2 was more conducive for root development in *W. chinensis*, but there was a non-significant effect on the growth of aboveground plant parts.

**TABLE 2:** Effect of different nitrogen forms and competitive treatments on plant growth under high nitrogen condition.

	Number of taproots	Number of lateral roots	Root length	Leaf area	Root/shoot ratio	Total biomass
A-S_1_	12 ± 1 abc	13 ± 1 abc	8.310 ± 0.400 abc	10.2632 ± 0.4878 b	0.1880 ± 0.0467 b	0.2897 ± 0.0178 abcd
B-S_1_	13 ± 1 abc	12 ± 4 abc	5.047 ± 1.254 bc	15.0722 ± 2.7449 a	0.0522 ± 0.0202 b	0.4168 ± 0.0691 a
C-S_1_	16 ± 1 ab	11 ± 3 bc	5.793 ± 0.723 abc	14.2742 ± 1.0921 a	0.0582 ± 0.0101 b	0.3228 ± 0.0659 ab
A-S_2_	8 ± 2 c	22 ± 3 a	10.214 ± 1.689 abc	3.7266 ± 0.4005 e	0.3930 ± 0.0839 a	0.1191 ± 0.0089 e
B-S_2_	12 ± 1 abc	14 ± 1 abc	4.579 ± 0.417 c	8.3448 ± 0.6154 bcd	0.0790 ± 0.0138 b	0.2178 ± 0.0499 bcde
C-S_2_	14 ± 1 abc	22 ± 2 ab	8.058 ± 1.154 abc	8.3858 ± 0.4256 bcd	0.1120 ± 0.0153 b	0.1993 ± 0.0342 bcde
A-MT	11 ± 3 abc	15 ± 6 abc	8.291 ± 1.339 abc	9.5289 ± 1.1631 bc	0.1754 ± 0.0154 b	0.2236 ± 0.0115 bcde
B-MT	13 ± 2 abc	14 ± 4 abc	11.556 ± 5.241 ab	13.7686 ± 1.0178 a	0.0680 ± 0.0121 b	0.3028 ± 0.0215 abc
C-MT	17 ± 1 a	11 ± 2 bc	7.390 ± 1.488 abc	16.4691 ± 1.2794 a	0.0750 ± 0.0281 b	0.3883 ± 0.0652 a
A-MC	13 ± 3 abc	21 ± 2 abc	11.861 ± 2.541 a	5.2247 ± 1.2043 de	0.5368 ± 0.1358 a	0.1810 ± 0.0448 cde
B-MC	13 ± 5 abc	11 ± 2 c	5.354 ± 0.485 abc	5.8742 ± 0.8165 cde	0.0701 ± 0.0020 b	0.1194 ± 0.0298 e
C-MC	9 ± 3 bc	16 ± 4 abc	6.897 ± 0.758 abc	6.9876 ± 0.5364 bcde	0.0920 ± 0.0071 b	0.1527 ± 0.0164 de

*S_1_, intraspecific competition of W. trilobata; S_2_, intraspecific competition of W. chinensis; MT, interspecific competition of W. trilobata; MC, interspecific competition of W. chinensis; A, no nitrogen addition; B, nitrogen form ratio AN:NN, 2:1 with total nitrogen amount of 15 g N⋅m^–2⋅^year^–1^; C, nitrogen form ratio AN:NN, 1:2 with total nitrogen amount of 15 g N⋅m^–2⋅^year^–1^; AA, ammonium nitrogen; NN, nitrate nitrogen. The values are means ± SE (n = 3). Different lowercase letters indicate significant differences between different treatments (p < 0.05).*

### Effect of Different N Forms and Competitive Treatments on Plant Photosynthesis

**FIGURE 3:**
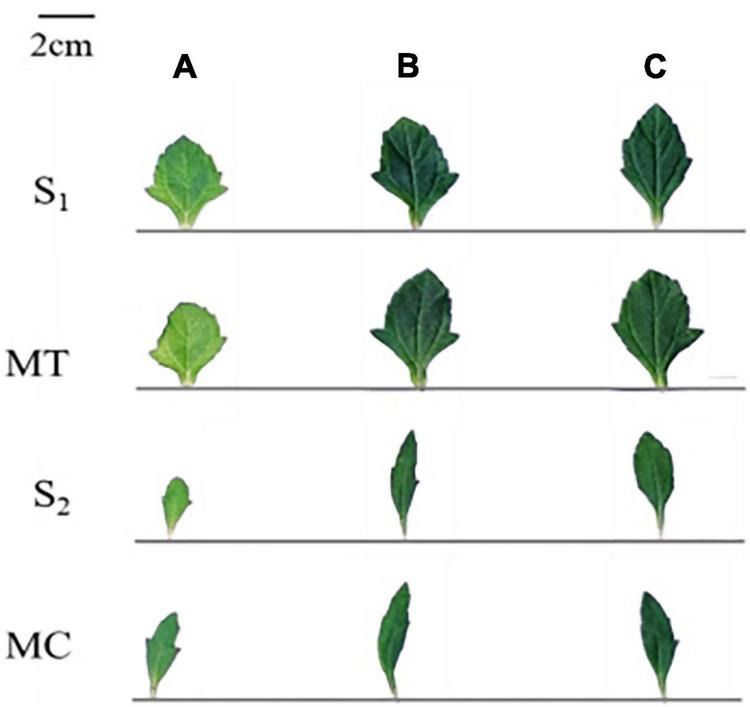
Leaf morphology of invasive *W. trilobata* and native *W. chinensis* under different N forms and competition period. AA, ammonium nitrogen; NN, nitrate nitrogen; S_1_, intraspecific competition of *W. trilobata*; S_2_, intraspecific competition of *W. chinensis*; MT, interspecific competition of *W. trilobata*; MC, interspecific competition of *W. chinensis*; **(A)** no nitrogen addition; **(B)** nitrogen form ratio AN:NN, 2:1 with total nitrogen amount of 15 g N⋅m^–2⋅^year^–1^; **(C)** nitrogen form ratio AN:NN, 1:2 with total nitrogen amount of 15 g N⋅m^–2⋅^year^–1^.

**FIGURE 4:**
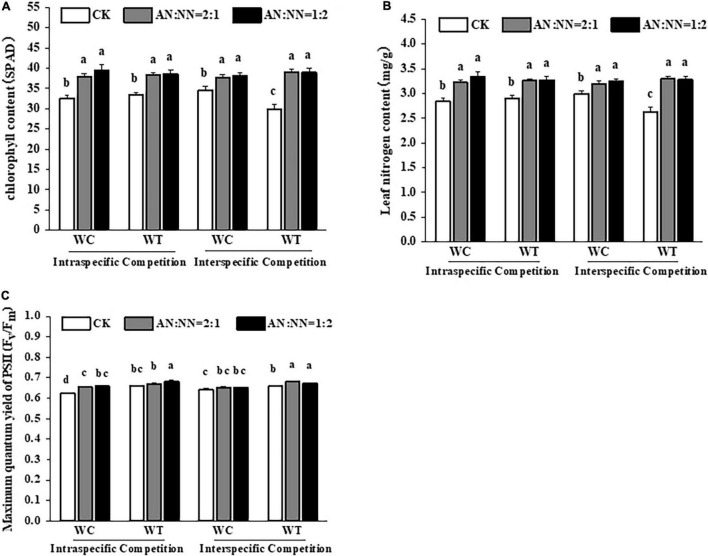
Effects of different N forms and competitive treatments on plant photosynthesis traits under high N conditions. WT, *W. trilobata*; WC, *W. chinensis*; CK, no nitrogen addition; AA, ammonium nitrogen; NN, nitrate nitrogen; N15(2:1), nitrogen form ratio AN:NN, 2:1 with total nitrogen amount of 15 g N⋅m^–2^⋅year^–1^; N15(1:2), nitrogen form ratio AN:NN, 1:2 with total nitrogen amount of 15 g N⋅m^–2^⋅year^–1^; **(A)** chlorophyll content; **(B)** leaf nitrogen content; **(C)** maximum quantum yield of PSII (mean ± SE, *n* = 3). Different lowercase letters above bars indicate significant differences between different treatments (*p* < 0.05).

The leaves of both species were greener under high N treatment than those of control treatment, indicating more chlorophyll content under the same N treatment (Figure 3). For both intraspecific and interspecific competition and N forms, the chlorophyll and leaf N contents of both species were significantly higher under high N treatment than that of the control treatment (Figures 4A,B).

Under high N treatment, regardless of intraspecific and interspecific competition, the *F*_*v*_/*F*_*m*_ values of both species were increased with a significant increase (*p* < 0.05) in *W. trilobata* compared with control treatment, while there was a non-significant increase for *W. chinensis* (Figure 4C). The effect of N forms on chlorophyll fluorescence was also non-significant.

### Effect of Different N Forms and Competitive Treatments on Plant Antioxidant System

**FIGURE 5:**
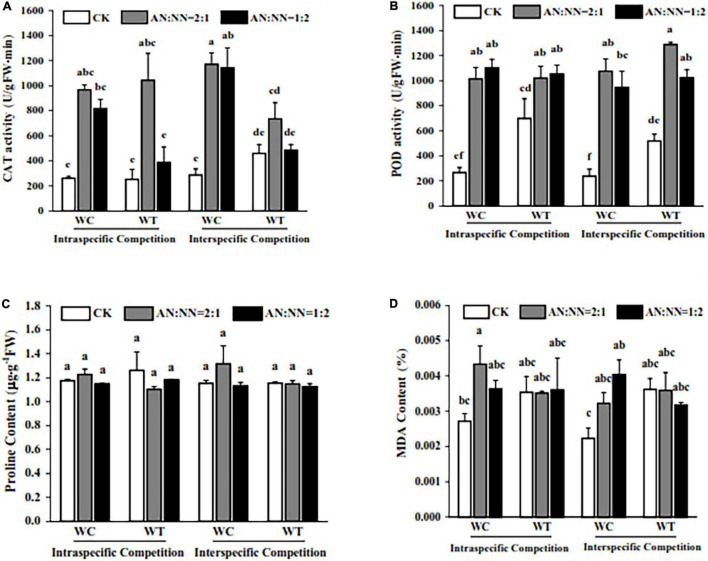
Effect of different N forms and competitive treatments on plant antioxidant system under high N condition. WT, *W. trilobata*; WC, *W. chinensis*; CK, no nitrogen addition; AA, ammonium nitrogen; NN, nitrate nitrogen; N15(2:1), nitrogen form ratio AN:NN, 2:1 with total nitrogen amount of 15 g N⋅m^–2^⋅year^–1^; N15(1:2), nitrogen form ratio AN:NN, 1:2 with total nitrogen amount of 15 g N⋅m^–2^⋅year^–1^; **(A)** catalase activity; **(B)** peroxidase activity; **(C)** proline content; **(D)** malondialdehyde content (mean ± SE, *n* = 3). Different lowercase letters above bars indicate significant differences between different treatments (*p* < 0.05).

Our data showed that high N significantly increased CAT activity; however, there was a non-significant difference in CAT activity for both species. For competition periods, interspecific competition increased CAT activity in both species with a significant increase in *W. trilobata*. Under high N treatment, CAT activity of *W. chinensis* was significantly increased than *W. trilobata*. For different N forms/ratios and species, CAT activity of *W. trilobata* was significantly increased under AN:NN = 2:1, (Figure 5A) but there was a non-significant difference for AN:NN = 1:2.

For POD activity, the addition of N significantly increased the POD activity in *W. trilobata* and *W. chinensis* compared with control treatment without N addition (Figure 5B). When we compared both species, POD contents in *W. trilobata* were significantly higher than *W. chinensis*. For the intraspecific competition, POD activity in *W. chinensis* had increased with a similar trend to that of *W. trilobata*; however, for interspecific competition, POD activity in *W. trilobata* was significantly higher than that of *W. chinensis*, particularly for N form of AN:NN = 2:1. For N forms and competition periods, POD activity of both species was higher under N treatment of AN:NN = 2:1 for intraspecific competition, while it was higher for N treatment of AN:NN = 1:2 during intraspecific competition. For MDA content, among species, *W. chinensis* significantly recorded lower content than that of *W. trilobata*. N forms had no significant effect on MDA content in *W. trilobata*, however, significantly affected the MDA content in *W. chinensis*. For competition periods and N forms, MDA contents were significantly increased under intraspecific competition with AN:NN = 2:1 and interspecific competition with AN:NN = 1:2 in *W. chinensis* than other competition periods and N forms. Our data also showed that compared with control, there was a non-significant difference for proline content in both species under high N treatment and competitive treatments, indicating that both high N and competition periods had no significant effect on proline content of both species, showing no resistance against stress (Figures 5C,D).

## Discussion

Nitrogen is an essential nutrient for plant growth and development, and its demands increased abundantly as compared with other elements (Toor et al., 2021). N deposition and its addition, sometimes, can increase the availability of N to plants, thereby promoting crop growth (Zhong et al., 2019). In this study, we found that the addition of N significantly promoted plant growth, which is consistent with previous studies of Liu et al. (2018) and Duan et al. (2019). We also found that plant leaves under N addition were greener than those of control treatment without N addition, similar to that of the findings of Keleher (2018). This may be due to that N addition increased N uptake and chlorophyll synthesis to promote the photosynthetic process (Moriwaki et al., 2019). Chlorophyll fluorescence kinetics technology is used to describe the mechanism of photosynthesis, which has been widely used by researchers in recent years. It can reflect the internal characteristics of plants by changing the related indicators (Bussotti et al., 2020). In this study, *F*_*v*_/*F*_*m*_ values were significantly increased under high N treatment, possibly because N absorption was conducive to photosynthesis in plants (Xu et al., 2020).

In recent years, most studies have focused on antioxidant defense systems mainly under heavy metals stress, including lead and aluminum (Malik et al., 2021) and iron (Wang X. et al., 2021). Nonetheless, still there is a lack of information on antioxidant activities under N stress. N has been reported to have beneficial and harmless effects on crop plants. However, some studies have found that high N can cause oxidative damage to plants, which is consistent with our results that excessive N had toxic effects on plants. Plants activate various defense mechanisms, including increasing antioxidant enzyme activities to enhance stress tolerance (Chu et al., 2021). We also found that the exotic plant *W. trilobata* had more adaptability to excessive N than *W. chinensis*, mainly through promoting its growth and antioxidant enzyme activities.

Invasive plants have a solid adaptive and competitive ability than native ones (Qin et al., 2013). When invasive plants are competing with their natives, they can expand and establish new populations within a short period of time to form survival communities (Sun et al., 2020), or produce allelochemicals to inhibit the growth of surrounding plants, so as to occupy the resources in the invasive site (He et al., 2011). In this study, we found that competitive treatments promoted plant growth. Among species, *W. trilobata* was more apparent than *W. chinensis*, because interspecific competition produces a competitive effect for resources, including light which is essential for photosynthesis (Shafiq et al., 2021). Under control treatment without N, the leaf color of *W. trilobata* was slightly lighter than that of *W. chinensis*. However, under high N treatment, *W. trilobata* had darker leaves than *W. chinensis*, mainly due to the competition among species to promote the efficient use of N by invasive *W. trilobata* to improve the photosynthetic efficiency.

Under competitive conditions, plants also undergo different changes in their biochemical attributes, including antioxidant activities (Riaz et al., 2021). In this study, we found that the activity of antioxidant enzymes in invasive *W. trilobata* was significantly increased during interspecies competition than the native one. Also, MDA content in *W. trilobata* was significantly decreased under the condition of interspecies competition than that of *W. chinensis*, in which a significant increase was recorded.

According to the degree of preference for two N sources, the plants can be divided into ammonium-loving (e.g., rice and potato) and nitrate-loving plants (e.g., tobacco, wheat, and vegetables). However, major studies have shown that N supply from mixed sources is more beneficial to plant growth than from a sole source (Fan et al., 2010; Zhao et al., 2021). Applying N in the form of combined NN and AN can alleviate the metabolic disorder caused by sole AN (Zhang et al., 2021). Some studies also reported higher photosynthetic rates when N was applied in the form of AN than that of NN (Song et al., 2007). However, some studies have suggested that higher rates of NN may inhibit the photosynthetic rates to a certain extent (Su et al., 2020), while some have found lower photosynthesis when the riparian plant was grown under AN than that of NN (Cai et al., 2021). In recent years, most studies have proposed that N application in the form of ammonium nitrate was more conducive to improving the photosynthetic rates and plant growth, which is consistent with the results of this experiment (Abbas et al., 2019). Plants showed different responses and preferences to different N forms, as reported by previously published reports (Yousaf et al., 2016a,b; Shah et al., 2017a,b, 2021a,b; Qian et al., 2021). However, in this study, we found that the difference in growth traits of the two species was not significant under the two N forms. It may be due to the fact that mixed N forms have a similar promoting effect on two species or due to the high N treatment.

## Conclusion

*Wedelia trilobata* significantly showed higher biomass under intraspecific competition compared with their natives *W. chinensis*. N addition significantly promoted the growth and antioxidant enzyme activities compared with the control treatment. For N forms, our results showed that AN/NN = 2:1 enhanced the enzyme activity, particularly in *W. chinensis*, as compared with AN/NN = 1:2. Among competition periods and tested species, MDA content was significantly decreased during the interspecific competition, particularly for *W. chinensis*, as compared with the intraspecific competition. We concluded that under competitive conditions, *W. trilobata* will adjust to high N conditions through better growth and an antioxidant defense system.

## Data Availability Statement

The raw data supporting the conclusions of this article will be made available by the authors, without undue reservation.

## Author Contributions

PH, FS, and DD conceived and designed the experiments. FS, HW, AA, YD, and DD analyzed the data. PH, FS, and AA wrote the manuscript. PH, FS, AA, and DD were involved in the related discussion. SH, TJ, and SA helped to improve the quality of the manuscript. All authors have read and agreed to the published version of the manuscript.

## Conflict of Interest

The authors declare that the research was conducted in the absence of any commercial or financial relationships that could be construed as a potential conflict of interest.

## Publisher’s Note

All claims expressed in this article are solely those of the authors and do not necessarily represent those of their affiliated organizations, or those of the publisher, the editors and the reviewers. Any product that may be evaluated in this article, or claim that may be made by its manufacturer, is not guaranteed or endorsed by the publisher.

## Footnotes

1
https://imagej.nih.gov/ij/
